# Supplementary material for: Mutations in the HBV PreS/S gene related to hepatocellular carcinoma in Vietnamese chronic HBV-infected patients
Source: PLoS One. 2022 Apr 7;17(4):e0266134. doi: 10.1371/journal.pone.0266134 (PMC8989215; doi:10.1371/journal.pone.0266134)
Supplement: S1 Table — (DOCX) [file pone.0266134.s001.docx]

**Table S1:** **Distributions of 6 mutations related to HCC in groups of personal and HBV characteristics (n=247)**

| **Mutation** | **Sex** | | **Age group** | | **HBeAg** | | **HBV DNA** (log_10-_copies/mL) | | **Genotype** | |
| --- | --- | --- | --- | --- | --- | --- | --- | --- | --- | --- |
|  | Male  (n=170) | Female (n=77) | <40 (n=104) | **≥40** (n=143) | Negative (n=105) | **Positive** (n=142) | <5 (n=42) | **≥5** (n=205) | B (n=135) | **C**  (n=110) |
| **W4P/R/Y (*PreS1*)** | 7 (4.1) | 3 (3.9) | 4 (3.8) | 6 (4.2) | 4 (3.8) | 6 (4.2) | 2 (4.8) | 8 (3.9) | 1 (0.7) | **9 (8.2) (p=0.022)^b^** |
| **F20S (*S*)** | 6 (3.5) | 0 | 1 (1) | 5 (3.5) | 4 (3.8) | 2 (1.4) | 1 (2.4) | 5 (2.4) | 2 (1.5) | 4 (3.6) |
| **T47A/E/V/K (*S*)** | 16 (9.4) | 7 (9.1) | 7 (6.7) | 16 (11.2) | 8 (7.6) | 15 (10.6) | 4 (9.5) | 19 (9.3) | 3 (2.2) | **20 (19.0) (p<0.001)^b^** |
| **P120S/T (*S*)** | 14 (8.2) | 7 (9.1) | 6 (5.8) | 15 (10.5) | **14 (13.3) (p=0.019)^a^** | 7 (4.9) | **8 (19) (p=0.013) ^b^** | 13 (6.3) | **21 (15.6) (p<0.001) ^b^** | 0 |
| **S174N (*S*)** | 3 (1.8) | 1 (1.3) | 1 (1) | 3 (2.1) | 1 (1) | 3 (2.1) | 1 (2.4) | 3 (1.5) | 1 (0.7) | 3 (2.7) |
| **P203R (*S*)** | 4 (2.4) | 4 (5.2) | 1 (1) | 7 (4.9) | 2 (1.9) | 6 (4.2) | 1 (2.4) | 7 (3.4) | 3 (2.2) | 5 (4.5) |
| ^a^ p value (*Chi square test*) ^b^ p value (*Fisher Exact test*) | | | | | | | | | | |
