# Supplementary material for: Mutations in the HBV PreS/S gene related to hepatocellular carcinoma in Vietnamese chronic HBV-infected patients
Source: PLoS One. 2022 Apr 7;17(4):e0266134. doi: 10.1371/journal.pone.0266134 (PMC8989215; doi:10.1371/journal.pone.0266134)
Supplement: S2 Table — (DOCX) [file pone.0266134.s002.docx]

Table S2: **Distribution of personal characteristics and HBV viral markers in HCC and non-HCC group** (n=247)

| **Characteristic** | | **CHB groups** | | **p^a^** |
| --- | --- | --- | --- | --- |
|  |  | **HCC (n= 49)** | **non HCC (n=198 )** |  |
| Sex | male | 41 **(83.7)** | 129 (65.2) | **0.012** |
|  | female | 8 (16.3) | 69 (34.8) |  |
| Age group | ≥40 | 42 **(85.7)** | 101 (51) | **<0.001** |
|  | <40 | 7 (14.3) | 97 (49) |  |
| HBeAg | positive | 19 (38.8) | 123 (62.1) | **0.003** |
|  | negative | 30 **(61.2)** | 75 (37.9) |  |
| HBV DNA  (log cps/mL) | < 5 | 23 (**46.9**) | 19 (9.6) | <0.001 |
|  | ≥ 5 | 26 (53.1) | 179 (90.4) |  |
| Genotype (n=245) | B | 25 (53.2) | 111 (56.1) | 0.72 |
|  | C | 22 (46.8) | 87 (43.9) |  |
| Fibrosis ≥F3 | Yes | 19 **(38.8)** | 38 (19.2) | **0.004** |
|  | No | 30 (61.2) | 160 (80.8) |  |

^a^ p value (*Chi square test*)
