# Supplementary material for: Mutations in the HBV PreS/S gene related to hepatocellular carcinoma in Vietnamese chronic HBV-infected patients
Source: PLoS One. 2022 Apr 7;17(4):e0266134. doi: 10.1371/journal.pone.0266134 (PMC8989215; doi:10.1371/journal.pone.0266134)
Supplement: S3 Table — (DOCX) [file pone.0266134.s003.docx]

Table S3: **Predictive values of point-mutations related to HCC** (n=247)

| **Point-mutations** | **Frequency** | **PPV** | **NPV** | **Sensitivity** | **Specificity** |
| --- | --- | --- | --- | --- | --- |
| **W4P/R/Y (*PreS1*)** | 4% | 60% | 81.9% | 12.2% | 98% |
| **T47A/E/V/K (*S*)** | 9.3% | 39.1% | 82.1% | 18.4% | 92.9% |
| **P120S/T (*S*)** | 8.5% | 38.1% | 81.9% | 16.3% | 93.4% |
| **S174N (*S*)** | 1.6% | 75% | 81.1% | 6.1% | 99.5% |
| **P203R (*S*)** | 3.2% | 50% | 81.2% | 8.2% | 98% |
