# Supplementary material for: Mutations in the HBV PreS/S gene related to hepatocellular carcinoma in Vietnamese chronic HBV-infected patients
Source: PLoS One. 2022 Apr 7;17(4):e0266134. doi: 10.1371/journal.pone.0266134 (PMC8989215; doi:10.1371/journal.pone.0266134)
Supplement: Attachment — Submitted filename: Response to reviewers letter.docx [file pone.0266134.s005.docx]

**Response to reviewers**

We would like to express our gratitude to the editors and reviewers of this manuscript for thorough review and providing us with highly constructive comments to improve it. We have revised the manuscript according to the suggestions and made appropriate changes where needed.

**Editor's comments:**

- Comments: We note that you have included the phrase “data not shown” in your manuscript. Unfortunately, this does not meet our data sharing requirements. PLOS does not permit references to inaccessible data. We require that authors provide all relevant data within the paper, Supporting Information files, or in an acceptable, public repository. Please add a citation to support this phrase or upload the data that corresponds with these findings to a stable repository (such as Figshare or Dryad) and provide and URLs, DOIs, or accession numbers that may be used to access these data. Or, if the data are not a core part of the research being presented in your study, we ask that you remove the phrase that refers to these data.

Reply: We removed the phrase “data not shown” in line 230, page 22, and replaced with data was shown completely in Table S2 "Distribution of personal characteristics and HBV viral markers in HCC and non HCC group". All relevant data are within the manuscript and its supporting information as File S2_Pres_S mutation related to HCC.xlxs.

- Comments: 4 independent reviewers together with editor reviewed this paper, all judged the importance of the findings obtained, however, the some major modifications are required especially, since S174N (S) point mutation showed the highest OR with significant P value, what are the sensitivity, specificity, PPV and NPV for this point mutation. It is also recommended to deep sequencing on some samples to see the rate of quasispecies of some of these significant mutations.

Reply: Regarding to the OR values of mutations on the final multivariable analysis, 2 *S* mutations (T47A/E/V/K(*S*) and P120S/T(*S*)) had revealed three fold increase in HCC risk and good confidential intervals. On the contrary, three remaining mutations had only been detected on small numbers of cases (W4P/R/Y(*preS1)* 3 cases, OR 11.56 (1.99-67.05); S174N(*S*) 4 cases, 29.73 (2.12-417.07); and P203R (*S*) 8 cases, OR 8.45 (1.43-50.06)) with especially high ORs (table 6). The wide 95% confidential intervals of OR with very high upper values of these intervals revealed that the sample size was not large enough for investigating these sporadic mutations. Ideally, the deep sequencing technique could potentially increase the detection rates due to its higher sensitivity. However, the cost effectiveness should be considered for the aims of studying these irregular mutations or investigating a larger population. We mentioned these explanations in line 350-360. Due to limited condition, we were unable to perform deep sequencing in this study to determine sensitivity, specificity, PPV and NPV for this point mutation S174N (S).

**Reviewer #1 's opinions:**

- Comments: The history of HB vaccination and HBsAb status of the studied patients are not presented here, indeed the occurrence of Pre-S/S gene mutations may be affected by both factors.

Reply: *Thank you for your great feedback.*

- During sample collection, the history of HB vaccination was not recorded in 212 CHB patients whose serum samples had been stored during 2014-2016. It was only recorded in 35 CHB patients that were recruited in 2020-2021. Thus, there was no sufficient data available in all patients that can be utilized to analyze the relation of the HB vaccination to Pre-S/S gene mutations.
- In line 294-299, we has clarified: "Moreover, among our study population, antiHBs that had been tested on 186 cases with clinical symptoms were tested antiHBs and had detected 37 cases (19.9%) with HBsAg-AntiHBs coexsistence, higher than the rates of 3-5% in other investigation. Therefore, it was presumed that CHB patients with varieties of presentations had been included in our study and contributed to the difference in rates of point-mutations compared to other studies".
- Comments: The duration of HBV infection of included patients and its relation to pre-S/S mutations as well as the HCC occurrence should be shown and analyzed.

Reply: Data from hospital electronic database cannot be used to directly respond to this question. Alternatively, the time from when patients had been diagnosed with CHB to the time of study participation was calculated using data from “year of CHB diagnosed” and “year of inclusion”.

- Comments: The method of liver fibrosis assessment is not mentioned in methodology, this should be clarified.

Reply: In line 117-123, it is added that "Cirrhosis was defined as having signs of portal hypertension (splenomegaly, ascites, vascular collaterals on abdominal wall, esophageal varices or portal hypertensive gastropathy on gastroscopy) and signs of insufficiency of liver function (palmar erythema, vascular spiders, low concentration of albumin (<35 g/dL), elevation of the international normalized ratio (INR >1.1), thrombocytopenia (<160,000/mm^3^)) as well as irregularity of hepatic surface on ultrasonography or F3 on Metavir score using Acoustic radiation force impulse (ARFI) measurement [22]". We based on the reference of "Udell JA, et al. Does this patient with liver disease have cirrhosis? JAMA. 2012;307(8):832-42".

- Comments: It would be of clinical interest; to present the relation between liver inflammation status on one hand (e.g., ALT, AST or grade of liver inflammation on liver biopsy if available) and the pre-s/s mutations as well as HCC occurrence on the other hand

Reply: In our data, we only used ALT cross-sectional data (one time at inclusion) and we didn't have information on liver biopsy. Relation between ALT (non repeated measurementy) and mutation and HCC were not in the scope of this study.

**Reviewer #2 's opinions:**

- Comments: My main concern is the value of generalising the presenting data which is limited to the Vietnamese population.

Reply: *Thank you for your comment.* With advantage of having decent number of patients in different stages of CHB enrolled in the study, our data can moderately represent the Vietnamese population. However, as the study was only conducted on patients with detectable HBV DNA, the rates of mutations are predicted to be possibly higher than general population. Therefore, in the scope of this study, we do not aim to generalize the frequencies of mutation. Instead, it is intended to determine the correlation between mutation of HBV on PreS/S genes and HCC in vietnamese population. Further studies are suggested to generalize data to chronic HBV infected HCC community.

**Reviewer #3 opinions:** *Many thanks for such addition*

- Comments: English editing is required

Reply: We edited academic English writing whole our manuscript.

- Comments: Please add the following paragraph in new Materials section
  Iine 35-35 page 8--> 247 CHB-infected patients were recruited, characterized by 68.8% males, 44.5% HBV genotype C, 54.4% HBeAg positive, 23.1% fibrosis score ≥F3 and 19.8% HCC
  Reply: It was summarized in the Result section.
- Comments: Please add major hydrophilic region line 39 page 8 (with point-mutation on major hydrophilic region (MHR) in abstract.

Reply: we edited “point-mutation on major hydrophilic region (MHR)” in abstract line 42.

- Comments: Please delete "We detected a wide spectrum of point-mutations on PreS/S regions in
  Vietnamese CHB patients” line 48 page 8

Reply: We removed that sentence and replaced with " We detected 61% amino acid changes on *PreS/S* regions in Vietnamese CHB patients" line 50-51, page 2.

- Comments: There are several of references are not according to the style of the journal
  Reply: we have completed a thorough review of all the references used in our study and made changes accordingly
- Comments: The presentation of the results is more complicated and it is better if authors represented it with more simple figures.

Reply: The tables are intended to describe the name of point-mutations along with their associated data for traceability purpose.

- Comments: In discussion: Please delete the subtitles in the discussion section, delete "Recently"
  Reply: all subtitles and "Recently" are deleted as per comment.

**Reviewer #4 's opinions:**

- Comments: Is there any significant correlation between the mutations identified in the multivariable analysis in table 6 and sex, age and HBeAg negative.

Reply: We have completed the data analysis and revised the result line 221-224 and Table S1: The characteristics of these 6 point mutations had been analysed and found that W4P/R/Y (*PreS1*) (p=0.022) and T47A/E/V/K (*S*) (p<0.001) had significant higher rates on genotype C; P120S/T (*S*) had higher rates on genotype B (p<0.001), HBeAg (-) group (p=0.019) and low HBV DNA group (<5 log copies/mL) (p=0.013) (Table S1).

- Comments: Since S174N (S) point mutation showed the highest OR with significant P value, what are the sensitivity, specificity, PPV and NPV for this point mutation. It is recommended to deep sequencing on some samples to see the rate of quasispecies of some of these significant mutations (as above).
- Comments: Tables 2,3 and 4 are very long, might move as supplementary tables.

Reply: Tables are kept as it to support the clarification of data collected and considered critical to overall results.

- Comments: In the abstract (at the end of third line of the results), it looks there are some information is missing here "and on PreS1 gene"

Reply: We have added in line 38-40: The most common point mutations included *N/H51Y/T/S/Q/P* (30.4%), *V68T* (44.9%), *T/N87S/T/P* (46.2%) on *PreS1* gene.

- Comments: Any abbreviations should be spelled out in the first place, such as MHR in the abstract.

Reply: "major hydrophilic region (MHR)" is added in line 42.

Sincerely,

Nguyen Thi Cam Huong

MD, PhD, Lecturer

Departments of Infectious diseases

University of Medicine and Pharmacy at Ho Chi Minh city
