# Supplementary material for: Mutations in the HBV PreS/S gene related to hepatocellular carcinoma in Vietnamese chronic HBV-infected patients
Source: PLoS One. 2022 Apr 7;17(4):e0266134. doi: 10.1371/journal.pone.0266134 (PMC8989215; doi:10.1371/journal.pone.0266134)
Supplement: Attachment — Submitted filename: Response to reviewers letter .docx [file pone.0266134.s006.docx]

**Response to reviewers**

We would like to express our gratitude to the editors and reviewers for recognizing our effort to improve our manuscript. We have revised the manuscript according to the suggestions and made appropriate changes where needed.

**Comments:** "The manuscript has been significantly improved, however, the authors failed to comply with the reviewer comment regarding the calculation of PPV, NPV, sensitivity and specificity using deep sequencing or their available current data. This would give a clinical significance for their findings”.

**Reply:** It has been described in line 239-243 of the result section: "S174N (S) had the highest OR (29.73) with positive predictive value (PPV), negative predictive value (NPV), sentitivity (SEN), specificity (SPE) for predicting HCC respectively, 75%, 81.1%, 6.1%, 99.5%. The predictive values of all other 4 point-mutations related to HCC were concretely described in *Table S3*, based on our current available data."

We explained more in line 354-372 of discussion section: "Regarding to the OR values of mutations on the final multivariable analysis, 2 *S* mutations including 23 cases of **T47A/E/V/K** (*S*) and 21 cases of **P120S/T** (*S*) had revealed three folds increase in HCC risk associated with reasonable confidential intervals. On the contrary, three remaining mutations had only been detected on small numbers of cases with especially high ORs and wide 95% confidential intervals including 3 cases of **W4P/R/Y** (*preS1)*, OR 11.56 (1.99-67.05); 4 cases of **S174N** (*S*)**,** OR 29.73 (2.12-417.07); and 8 cases of **P203R** (*S*), OR 8.45 (1.43-50.06)) (table 6). A small sample size of this study resulted in a wider confidence interval with a larger margin of error for these sporadic mutations. It was suggested that a tighter confidence interval with values closer to the actual OR would be obtained if the sample size was increased. We had calculated the predictive values of these 5 point-mutations and had all found the high SPE and NPV values, but all revealed modest SEN due to small number of cases. S174N (*S*) for instance had been observed in our study with the highest OR and relative high PPV, NPV and specificity (75%, 81.1% and 99.5 %, respectively) but its sensitivity was only 6.1% (Table S3). If possible, the deep sequencing technique with its higher sensitivity could either potentially increase detection rates or improve the SEN values of these low-frenquency point-mutations. However, we were unable to perform it this time due to a large cost associated with the technique. Further studies are recommended in continuing upon findings of this study in which the direct sequencing would be the best and compulsory technique for better recognizing point mutations at quacispecies levels."

Sincerely,

Nguyen Thi Cam Huong

MD, PhD, Lecturer

Departments of Infectious diseases

University of Medicine and Pharmacy at Ho Chi Minh city, Vietnam
